# Dissecting the functional behavior of the differentially phosphorylated prolyl isomerase, Pin1

**DOI:** 10.1002/pro.5138

**Published:** 2024-08-16

**Authors:** Danielle F. Kay, Adem Ozleyen, Cristina Matas De Las Heras, Richard G. Doveston, Aneika C. Leney

**Affiliations:** ^1^ School of Biosciences University of Birmingham Edgbaston UK; ^2^ Leicester Institute of Structural and Chemical Biology University of Leicester Leicester UK; ^3^ School of Chemistry University of Leicester Leicester UK

**Keywords:** native mass spectrometry, phosphorylation, post‐translational modifications, prolyl isomerase, proteomics

## Abstract

Protein post‐translational modifications (PTMs) play an intricate role in a diverse range of cellular processes creating a complex PTM code that governs cell homeostasis. Understanding the molecular build‐up and the critical factors regulating this PTM code is essential for targeted therapeutic design whereby PTM mis‐regulation is prevalent. Here, we focus on Pin1, a peptidyl‐prolyl *cis‐trans* isomerase whose regulatory function is altered by a diverse range of PTMs. Through employing advanced mass spectrometry techniques in combination with fluorescence polarization and enzyme activity assays, we elucidate the impact of combinatorial phosphorylation on Pin1 function. Moreover, two phosphorylation sites were identified whereby Ser71 phosphorylation preceded Ser16 phosphorylation, leading to the deactivation of Pin1's prolyl isomerase activity before affecting substrate binding. Together, these findings shed light on the regulatory mechanisms underlying Pin1 function and emphasize the importance of understanding PTM landscapes in health and disease.

## 1 INTRODUCTION

Protein post‐translational modifications (PTMs) play vital roles in signal transduction, protein trafficking, cell proliferation, and cell differentiation. PTMs regulate protein conformation, localization, stability, and protein activity, and are tightly regulated to maintain cell homeostasis. An intricate combination of PTMs creates a complex “PTM code” (Lothrop et al., 2013; Minguez et al., 2013) which can be recognized by “reader” proteins leading to a physiological effect (Venne et al., 2014). Unraveling the PTM code is a major challenge. Overcoming this challenge requires technology that can both detect combinations of PTMs on single proteins, locate the PTM sites, and monitor how each individual PTM alters its protein function and the interplay with its surrounding proteins. PTMs also alter the function of “readers,” “writers,” and “erasers” within the PTM machinery itself. One important example is the peptidyl‐prolyl isomerase, Pin1, which is tightly regulated by phosphorylation.

Pin1 recognizes phosphorylated Ser/Thr‐Pro motifs within substrate proteins and catalyzes the *cis/trans* isomerization of the proline residue, leading to conformational changes within the substrate that constitute a PTM in their own right and strongly influence cell signaling, growth, and proliferation (Crenshaw et al., 1998; Fujimori et al., 1999; Lee, Pastorino, & Lu, 2011; Lu & Zhou, 2007; Shen et al., 1998; Wulf et al., 2001). Pin1 comprises two domains: a N‐terminal WW domain (residues 1–39) and a C‐terminal peptidyl‐prolyl *cis/trans* isomerase (PPIase) domain (residues 50–163) (Ranganathan et al., 1997). The WW domain binds specifically to the phosphorylated Ser/Thr‐Pro motifs, while the PPIase domain catalyzes prolyl *cis/trans* isomerization (Lu et al., 1999).

Pin1 itself is regulated by a combination of PTMs that remains to be fully deciphered (Chen et al., 2020). Phosphorylation plays a particularly important role, and hyperphosphorylation of Pin1 has been observed in Alzheimer's disease (Ando et al., 2013) and cancer (Lee, Chen, et al., 2011; Rangasamy et al., 2012; Wiegand et al., 2009; Wulf et al., 2001). Pin1 is phosphorylated at Ser16 within the WW‐domain by a number of kinases, including cAMP‐dependent protein kinase (PKA), Aurora A, Ribosomal protein S6 kinase 2 (RSK2), and MAP3K‐related serine/threonine kinase (COT), all of which lead to different downstream effects. Pin1 Ser16 phosphorylation by PKA and Aurora A disrupts its substrate binding ability to MPM‐2 antigens (Lu et al., 2002) and Bora (Lee et al., 2013), respectively. Conversely, Pin1 Ser16 phosphorylation by RSK2 and COT promotes 2‐O‐tetradecanoylphorbol‐13‐acetate‐induced cell transformation (Cho et al., 2012) and mammary gland tumorigenesis (Kim et al., 2015), respectively. Pin1 also has phosphorylation sites within the PPIase domain: Ser65 (Eckerdt et al., 2005), Ser71 (Lee, Chen, et al., 2011), Ser138 (Rangasamy et al., 2012), and Ser115 (Lepore et al., 2021). Pin1 phosphorylation at Ser65 by Polo‐like kinase 1 (Plk1) (Eckerdt et al., 2005), and at Ser115 by c‐Jun N‐terminal kinases (JNK) (Lepore et al., 2021), does not alter Pin1 catalytic activity but enhances Pin1 stability by preventing its ubiquitination and subsequent degradation (Eckerdt et al., 2005; Lepore et al., 2021). Phosphorylation at Ser138 by mixed‐lineage kinase 3 (MLK3) is also an example of positive regulation. This leads to increased Pin1 catalytic activity and nuclear localization which drives cell cycle progression and centrosome amplification (Rangasamy et al., 2012). In contrast, Ser71 phosphorylation by the death‐associated protein kinase 1 (DAPK1) and PKA inactivates Pin1 isomerase activity, prevents its nuclear localization, and inhibits its function as a cell‐cycle regulator (Lee, Chen, et al., 2011). These findings underscore the intricate regulation that phosphorylation has on Pin1 and the importance of precisely determining the combinatorial effect PTMs could have on Pin1 function.

Mass spectrometry (MS) is a powerful tool used to investigate protein PTMs and the PTM code (Bagwan et al., 2021; Leutert et al., 2021). By analyzing proteins intact, the identity and stoichiometry of PTMs can be determined. In addition, through top‐down (Brodbelt, 2022; Habeck & Lermyte, 2023) or bottom‐up fragmentation techniques, PTM sites can be localized. Moreover, by combining fragmentation‐based MS approaches with native MS (Leney & Heck, 2017; Tamara et al., 2022), the analysis of proteins in their non‐denatured state, information on how each step‐wise PTM alters the structure and function of the modified protein can be elucidated (Ben‐Nissan et al., 2017; Drepper et al., 2021; Lössl et al., 2016). Here, we utilize this technology to determine the sequence of events behind Pin1 inactivation upon phosphorylation by PKA. Using fluorescence polarization (FP) and colorimetric PPIase activity assays, we show phosphorylation of Pin1 impacts both substrate recognition and Pin1's ability to isomerize pSer/Thr‐Pro motifs. Native MS data further showed that two sites underwent phosphorylation by PKA. Moreover, by combining native MS and bottom‐up proteomics data, these phosphosites were localized on the WW and PPIase domains, the order of phosphorylation site occupancy determined, and their individual impact on Pin1 activity elucidated. The data systematically illustrate how phosphorylation of Pin1 alters its ability to “read” and “write” the PTM code. In turn, this highlights the importance of understanding the combinatorial impact of Pin1's PTMs for its emergence as a target for therapeutic intervention.

## 2 RESULTS AND DISCUSSION

### 2.1 Pin1 phosphorylation regulates its ability to bind substrates and catalyze prolyl isomerization

**FIGURE 1:**
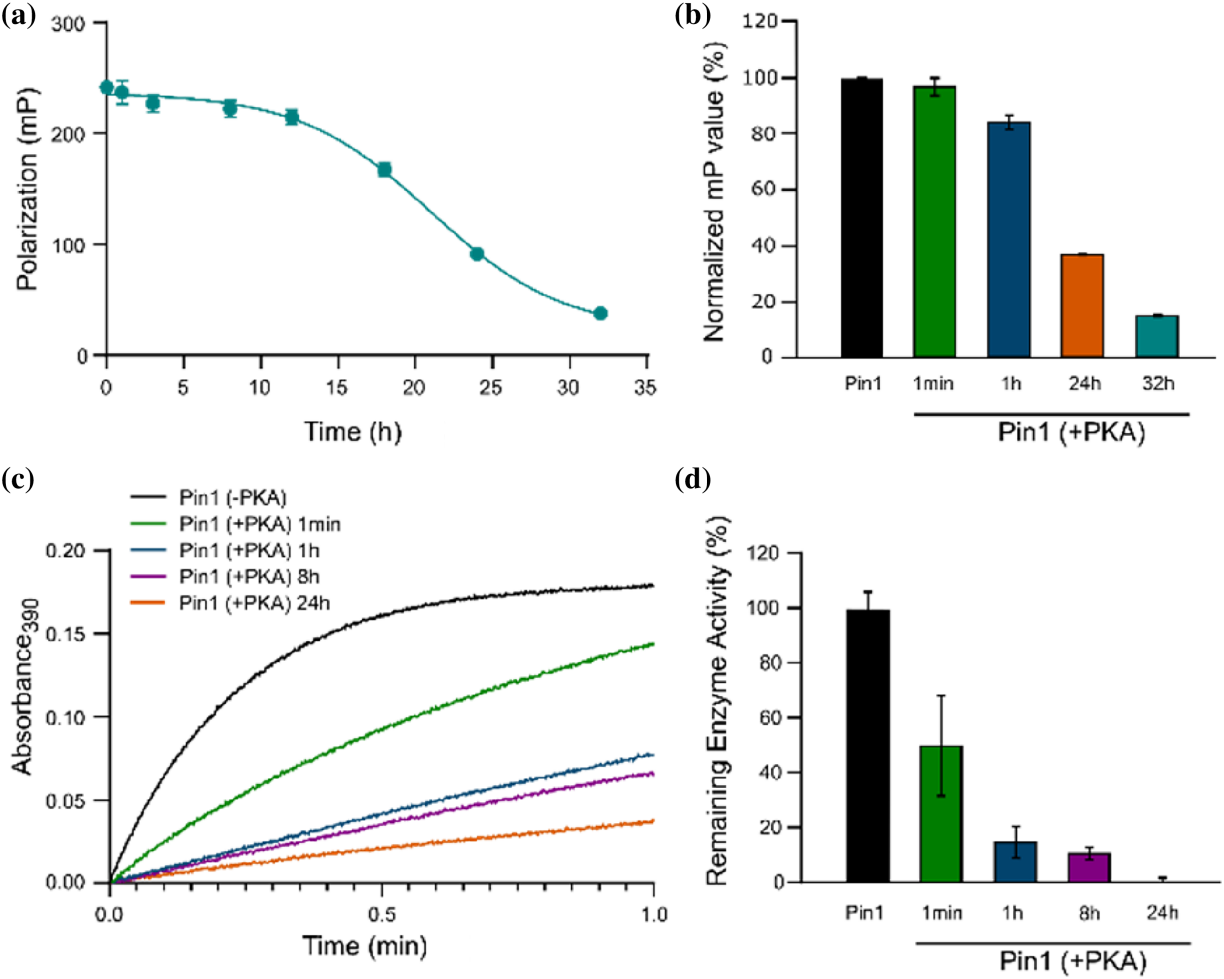
Phosphorylation prevents Pin1‐substrate binding and inhibits enzyme activity. Pin1 was incubated with cAMP‐dependent protein kinase (PKA) for the indicated times prior to all analysis. (a) Time‐dependent fluorescence polarization (FP) analysis of FITC‐Pintide binding to Pin1 in response to PKA phosphorylation. The data was fitted for illustrative purposes to a variable‐slope sigmoidal least squares model in GraphPad Prism. (b) PKA‐induced % change in polarization over time (green, blue, and red) relative to a control in the absence of PKA (black). (c) Chymotrypsin‐coupled spectroscopic assay measuring change in absorbance at 390 nm as a function of time after treatment of the suc‐AEPF‐pNA with Pin1, whereby absorbance is proportional to Pin1 enzymatic activity. (d) Relative remaining % enzymatic activities after 0.5 min following treatment of suc‐AEPF‐pNA with Pin1. All data was collected in triplicates and presented as the mean ± standard error of the mean.

Phosphorylation of Pin1 by PKA at Ser16 was previously shown to prevent binding of Pin1 substrates to its WW‐domain (Lu et al., 2002; Smet‐Nocca et al., 2013). To confirm this, the effect of PKA‐induced Pin1 phosphorylation on its affinity for a substrate was assessed using a time dependent FP assay. “Pintide” (WFYpSPFLE), a synthetic peptide optimized for high affinity binding to the WW‐domain (Lu et al., 1999) was selected as a model substrate and fluorescently labeled at the N‐terminus (apparent *K*_d_ for Pin1 binding = 0.7 μM, Figure S1a). As expected, binding of ‘Pintide’ to Pin1 was inhibited in the presence of PKA in a time‐dependent manner, consistent with the expected effect of Ser16 phosphorylation (Figures 1a,b and S1b). Notably, this time‐dependent PKA‐induced decrease in polarization was slow: only 16% of binding was inhibited after 1 h, with full inhibition observed only after 32 h (Figure 1a,b).

In addition to Ser16 phosphorylation, PKA is known to phosphorylate Pin1 at Ser71, leading to loss of PPIase catalytic activity (Lee, Chen, et al., 2011). To investigate this, we used a chymotrypsin‐coupled spectroscopic assay (Lu et al., 1999; Smet‐Nocca et al., 2013). In this experiment, an artificial substrate peptide suc‐AEPF‐pNA preferentially adopts a *cis*‐proline conformation due to the presence of trifluoroethanol and LiCl. Upon isomerization to the *trans*‐prolyl conformation, the p‐nitroanaline (pNA) chromophore is selectively cleaved from the peptide by chymotrypsin, resulting in an increase in absorption at 390 nm (Lu et al., 1999; Smet‐Nocca et al., 2013). As expected, in the absence of PKA, absorption at 390 nm increased upon the addition of Pin1 to the substrate, thereby confirming Pin1 catalytic activity (Figure 1c,d). Upon the introduction of PKA, this activity decreased in a time‐dependent manner that was much faster than that observed in the FP binding assay: 85% of Pin1 catalytic activity inhibited after only 1 h (Figure 1c,d). This result was intriguing because it indicated that treatment of Pin1 with PKA led to fast inhibition of catalytic activity but much slower inhibition of WW‐domain optimized substrate binding.

### 2.2 Two phosphorylation sites on Pin1 detected by native MS

**FIGURE 2:**
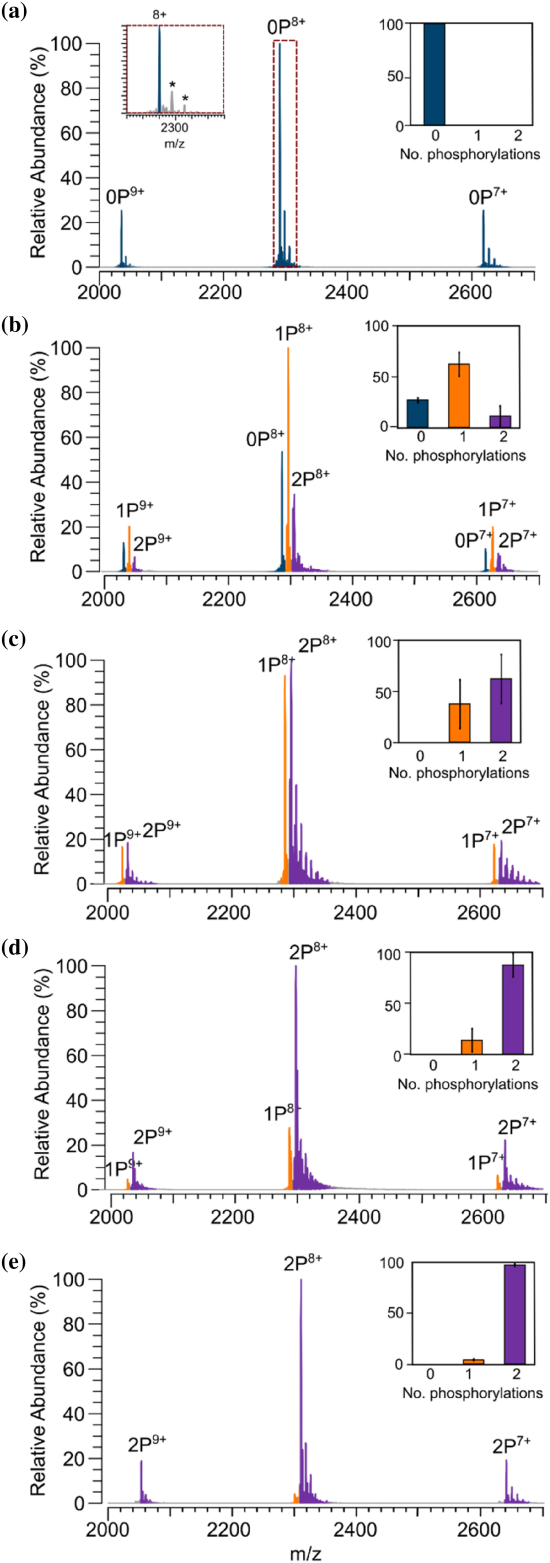
Native mass spectrometry identifies up to two phosphorylation sites on Pin1. Pin1 was incubated in the absence (a) and presence (b–e) of cAMP‐dependent protein kinase (PKA) for 1 h (b), 3 h (c), 8 h (d), and 24 h (e). Unmodified Pin1 (dark blue), singly phosphorylated Pin1 (orange), and doubly phosphorylated Pin1 (purple) are highlighted. Bar charts show the average relative abundance of each Pin1 proteoform. The data were collected in triplicate, with the error reported as the standard deviation. * Represents ~60–64 Da adducts.

We hypothesized that PKA might differentially phosphorylate multiple sites on Pin1 in a time‐dependent manner. To address this, native MS was first used to confirm if PKA was phosphorylating multiple sites on Pin1. Thus, Pin1 was treated with PKA for different time periods, and the extent of phosphorylation was determined based on observed mass changes to Pin1. Prior to phosphorylation, native MS showed a single, narrow charge state distribution corresponding to Pin1 with a measured mass of 18,312 Da (Figure 2a, Table S1). After incubation of Pin1 with PKA for 1 h, a second series of peaks dominated the spectrum with a mass shift of 80 Da representative of a single phosphorylation site (Figure 2b). Interestingly, at 3 and 8 h, peaks corresponding to doubly phosphorylated Pin1 were observed at a relative abundance of ~65 ± 23% and 85% ± 11%, respectively (Figure 2c,d). Finally, at 24 h, peaks corresponding to two Pin1 phosphorylation sites dominated the spectrum (95% ± 0.01%), with no further phosphorylation sites detected (Figure 2e).

### 2.3 Phosphorylation sites localized to both PPIase and WW‐domains

**FIGURE 3:**
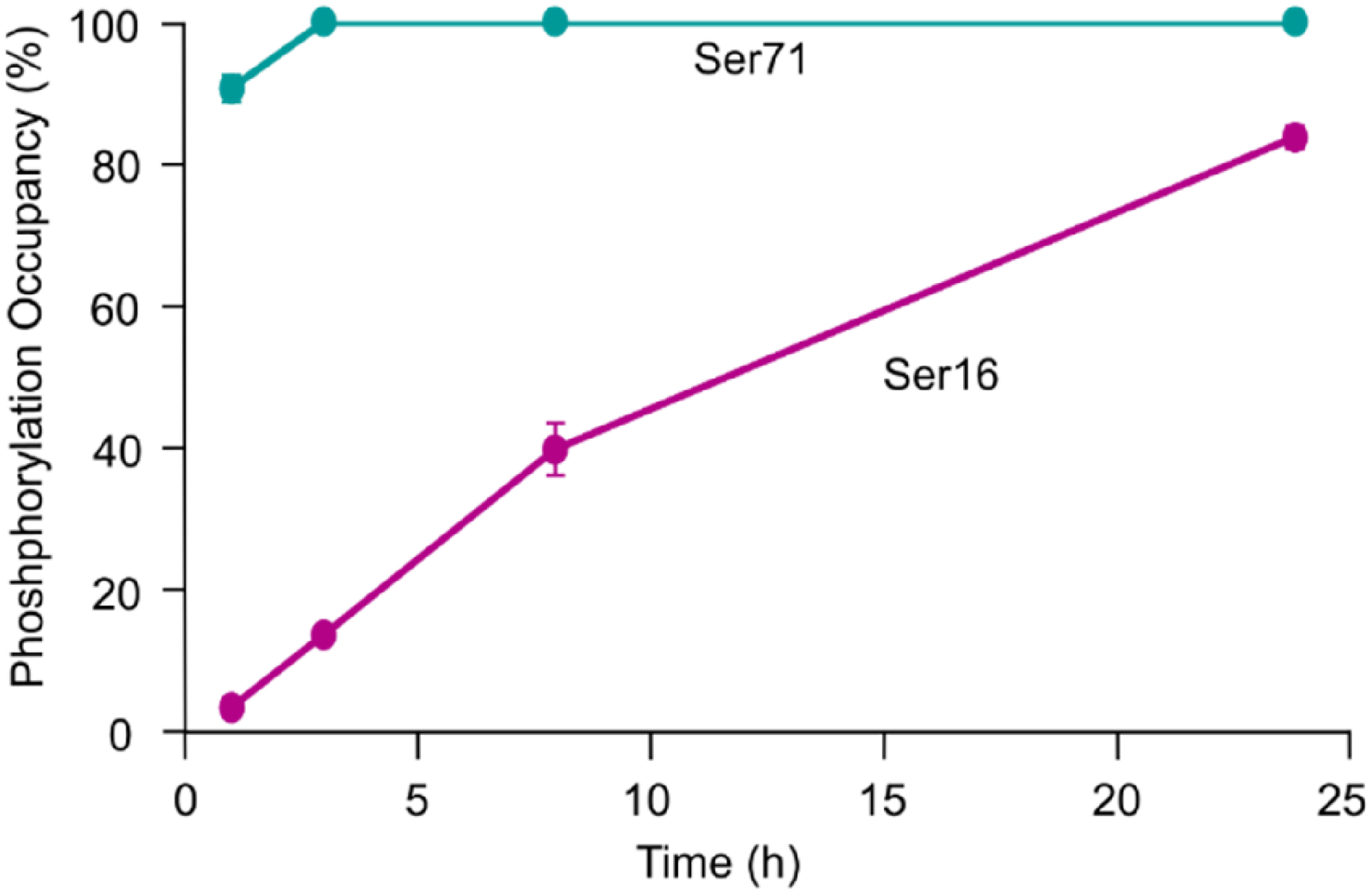
Liquid chromatography–Mass spectrometry/Mass spectrometry (LC–MS/MS) data localizes time‐dependent phosphorylation sites on Pin1. Phosphorylation occupancy of Ser71 and Ser16 containing peptides upon incubation with protein kinase A, whereby 100% phosphorylation occupancy corresponds to one phosphorylation site. The data were collected in triplicate with the error reported as the standard deviation.

To localize the sites of phosphorylation, differentially phosphorylated Pin1 was digested with trypsin and the resulting peptides analyzed by liquid chromatography (LC) MS/MS. Two predominant phosphosites were detected at Ser16 (Figure S2) and Ser71 (Figure S3). We noted that miscleavage sites were observed around the Ser71 and Ser16 phosphosites upon digestion with trypsin. Thus, to aid quantification of the Ser16 and Ser71 phosphopeptides, the endopeptidase Lys‐C was used. Comparing, the phosphorylation occupancy of the Ser16 and Ser71 phosphopeptides at each time‐point shows that ~90% Ser71 is phosphorylated after 1 h (Figure 3, Tables S2 and S3, Figure S4). In contrast, the abundance of Ser16 phosphopeptide compared to its unphosphorylated counterpart was much lower (4%), with its abundance only slowly increasing to ~80% after 24 h of incubation with PKA (Figure 3, Tables S2 and S3, Figure S4). Lower abundant phosphorylation sites at Ser58, Ser65, and Ser67 were detected (Table S4), however, these were not consistently detected across all timepoints, indicating they are not the predominant sites of PKA phosphorylation.

### 2.4 Time‐dependent phosphorylation sequentially alters Pin1's ability to recognize its substrates

**FIGURE 4:**
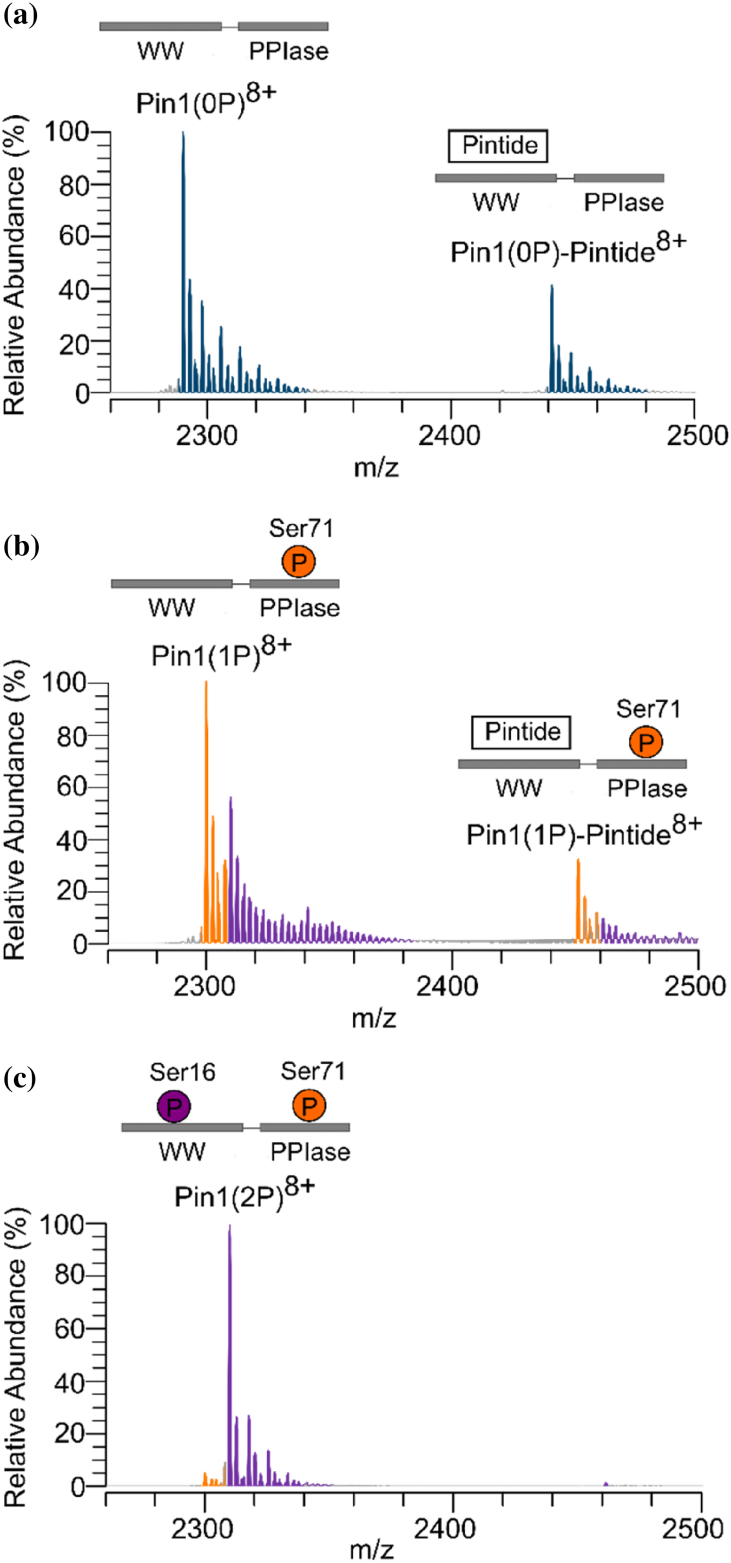
Native Mass spectrometry (MS) show impact of phosphorylation on Pin1 substrate recognition. Native MS of Pintide incubated with (a) unmodified Pin1 and Pin1 after phosphorylation with protein kinase A for (b) 3 h and (c) 24 h. Peaks corresponding to the 8+ charge state of unmodified Pin1, singly phosphorylated Pin1, and doubly phosphorylated Pin1 are highlighted in blue, orange, and purple, respectively.

Our LC–MS/MS data highlights the time‐dependent nature of Pin1 phosphorylation with Ser71 phosphorylation preceding Ser16 phosphorylation. To determine precisely how these phosphorylation sites alter Pin1's function, Pin1 was phosphorylated at Ser71 and at Ser16 in combination with Ser71, and its ability to bind Pintide was probed using native MS (Figure 4, Figure S5, Table S5). Prior to phosphorylation, peaks were present corresponding to Pin1 and a Pin1‐Pintide complex consistent with Pintide's known affinity for the WW‐domain on Pin1 (Lu et al., 1999). When Ser71 was phosphorylated, a small, yet significant, difference in Pin1 binding to Pintide was observed (Figure 4b, Table S5), consistent with low quantities of Ser16 detected by LC–MS/MS (Figure 3). Finally, upon dual phosphorylation of Pin1, the Pin1‐Pintide complex was unable to form (Figure 4c). Together, the native MS functional assay provides further support that phosphorylation occurs in a sequential manner whereby only once Ser16 is phosphorylated, Pin1 is prevented from binding its substrates.

## 3 CONCLUSIONS

**FIGURE 5:**
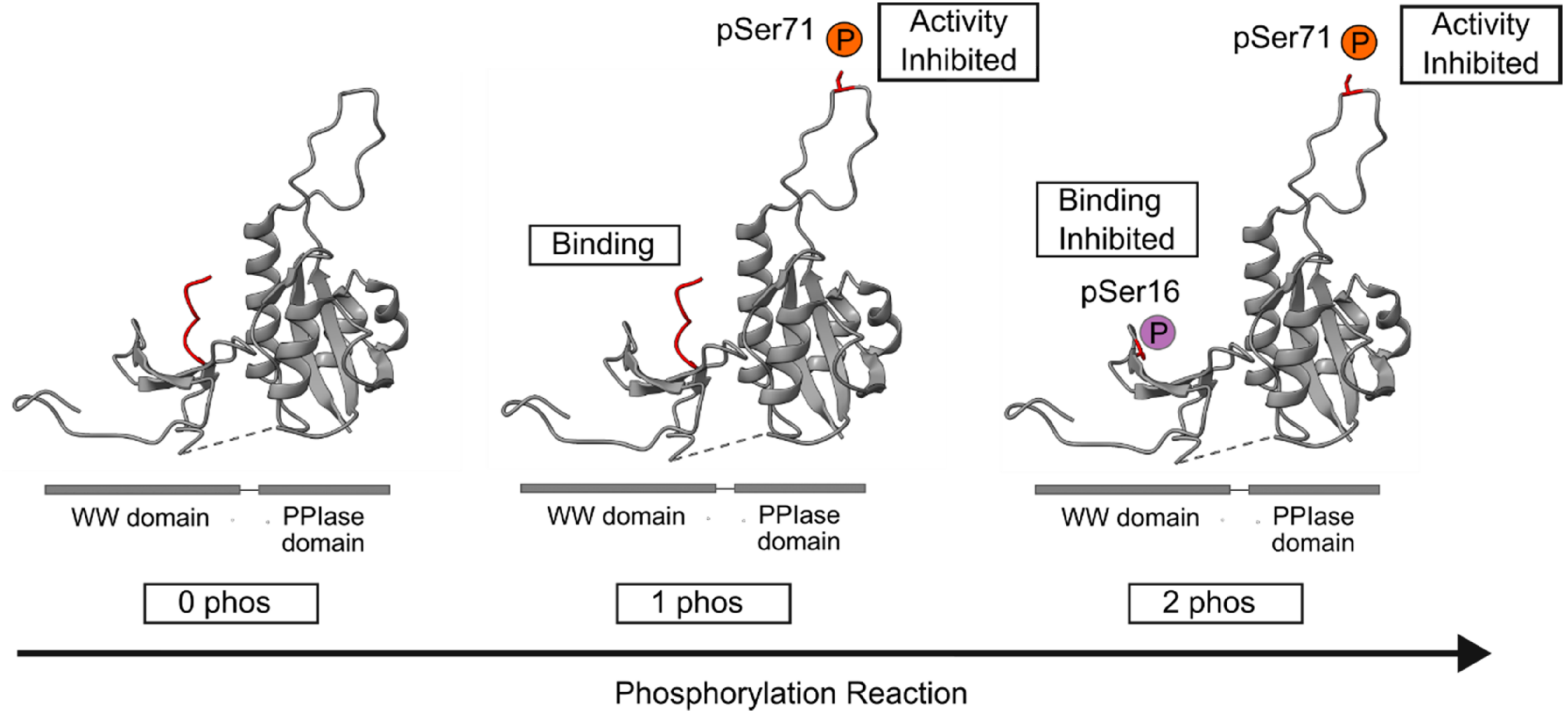
Schematic of phosphorylation impact on Pin1's function. Timeline of phosphorylation highlighting sites identified (orange and purple) and their resulting impact on Pin1 binding and catalysis. A phosphopeptide indicative of where substrates bind the WW domain is highlighted in red (PDB code: 1F8A).

Increasing fundamental knowledge on how Pin1 is regulated via combinatorial PTM is critical for accelerating the maturation of Pin1 as a therapeutic target. Moreover, 15 phosphorylation sites have been suggested to occur on Pin1 (Hornbeck et al., 2015), many of which likely co‐occur when multiple kinases are present in vivo. Here, we use a model kinase, PKA, to show that Pin1 can be simultaneously phosphorylated at two sites, Ser16 and Ser71. In contrast to previous studies that focused primarily on Ser16 phosphorylation (Lu et al., 2002; Smet‐Nocca et al., 2013), we observe Ser71 to be the primary phosphorylation site (Figures 3 and 5). This is supported by NMR studies of the Pin1 S71E phosphomimetic mutant that showed S71 was evolutionarily primed to respond to modification and whose effects propagate across the Pin1 catalytic loop (Mahoney et al., 2018). We also show that Pin1's function is deactivated in a sequential manner whereby PPIase activity is inhibited first upon Ser71 phosphorylation (Figures 1 and 3), followed by substrate binding following Ser16 phosphorylation (Figures 1, 3, 4, and 5).

Phosphorylation of the PPIase domain prior to the WW domain is intriguing because it suggests Pin1 can exist in an intermediate state whereby it can recruit substrates but not catalyze their *cis‐trans* prolyl isomerization. Indeed, this data supports a dual binding model for Pin1, whereby Pin1 recruits substrates through both the WW domain and the PPIase domain. Substrate isomerization could therefore occur at the bound proline, or a proximal one (Innes et al., 2013). This may also point to Pin1 having a role as a “hub” protein that integrates protein–protein interaction networks, in addition to its role as a prolyl isomerase enzyme. Dual phosphorylation could, therefore, act as a “failsafe” mechanism for Pin1 inhibition, which could be exploited as a point for therapeutic intervention in Pin1‐related disease.

Together, our work highlights the use of a combinatorial native and bottom‐up MS approach to not only identify PTM sites, but also correlate PTM site occupancy with protein substrate recruitment and activity. Through advances in top‐down MS methodology and data analysis, this type of analysis could be performed within a single MS experiment. Indeed, our previous work has shown extensive fragmentation coverage of Pin1 using top‐down MS and additionally revealed phosphopeptide binding sites on Pin1 using a MS3 approach (Tamara et al., 2017). Moreover, considering many proteins contain multiple phosphorylation sites and have been shown to bind their interaction partners in a phosphorylation‐specific manner, we anticipate our approach will become widely applicable for studying the impact of the PTM code on proteoform‐specific biomolecular interactions.

## 4 MATERIALS AND METHODS

### 4.1 Pin1 expression and purification

Plasmid DNA (ampicillin resistance) encoding for His_6_‐Pin1 was isolated from an XL1 Blue *E. coli* stab culture obtained as a gift from Dustin Maly (Addgene #40773; http://n2t.net/addgene:40773; RRID:Addgene_40773). The stab culture was plated onto Luria broth (LB) agar containing 100 μg/mL ampicillin and incubated at 37°C overnight. A single colony was used to inoculate 5 mL of terrific broth (TB) containing 100 μg/mL ampicillin and 0.4% (v/v) glycerol. This culture was shaken for 18 h at 37°C. Plasmid DNA was isolated from the bacterial cells using an E.Z.N.A.® Plasmid DNA Mini Kit I (#D6942‐01, omega BIO‐TEK).

The plasmid was transformed into BL21 (DE3) *E. coli* cells (New England BioLabs) using the heat shock method. The transformed cells were plated onto LB agar containing 100 μg/mL ampicillin and incubated overnight at 37°C. A single colony was used to inoculate 500 mL of TB containing ampicillin (100 μg/mL). The culture was incubated at 37°C to an optical density (OD) of 0.7. Then, overexpression of His_6_‐Pin1 was induced by the addition of isopropyl b‐D‐1‐thiogalactopyranoside (IPTG, 0.4 mM) and the cultures were incubated at 25°C for 18 h. The cells were harvested by centrifugation and suspended in 15 mL of lysis buffer (50 mM HEPES, 300 mM NaCl, 1.0 mM DTT, pH 7.4), containing DNAase, a Pierece™ protease inhibitor (5 mg per pellet), and 5 mg of lysozyme. The cells were lysed by sonication and the cleared lysates obtained by centrifugation.

The His_6_‐Pin1 protein was isolated by gravity flow Ni^2+^‐affinity chromatography with elution using the lysis buffer (see above) containing increasing concentrations of imidazole (25–500 mM). The fractions containing His_6_‐Pin1 were pooled and dialyzed against protein storage buffer containing 50 mM HEPES, 300 mM NaCl, 1.0 mM DTT, 10% (v/v) glycerol, and pH 7.4 using a dialysis membrane (3.5 K MW cut off). The His_6_‐Pin1 protein solution was then concentrated using a 10 kDa cut‐off centrifugal filter unit (Merck Millipore) and stored at −80°C. To remove the His tag, His_6_‐Pin1 was treated with TEV protease (molar ratio of 50:1) and incubated overnight at 4°C. Pin1 was purified from TEV and His_6_ by Ni^2+^‐affinity chromatography column as described previously, whereby Pin1 was obtained from the flow through. The Pin1 protein solution was dialyzed against protein storage buffer, concentrated, and stored as described above.

### 4.2 Pin1 phosphorylation for biophysical assays

To perform the FP and enzymatic activity assays, Pin1 was phosphorylated by incubation of 40 μM Pin1 with 10,000 units of cAMP‐dependent protein kinase (PKA) (New England BioLabs) in kinase buffer (50 mM Tris‐HCl, 10 mM MgCl_2_, 1 mM DTT, and pH 7.5) supplemented with 2 mM adenosine triphosphate (ATP) (New England BioLabs) at 37°C. At 1 min, 1 h, 3 h, 8 h, 12 h, 18 h, 24 h, and 32 h, aliquots were taken, and PKA activity was stopped by addition of 20 mM EDTA, and the samples were kept at −80°C prior to analysis.

### 4.3 Fluorescence polarization assay

FP experiments were conducted using a fluorescein (FITC)‐labeled “Pintide” [5‐FITC‐(Ahx)‐WFY(pS)PFLE‐CONH_2_] purchased from China Peptides. FP data were collected at room temperature in buffer containing 5 mM HEPES pH 7.4, 30 mM NaCl, 0.1% v/v Tween20, and 1% v/v DMSO using Corning black, round‐bottom, low‐binding 384‐well plates. For all experiments, a fixed concentration of 10 nM fluorescently labeled Pintide and 40 μM of Pin1 protein was used. All plates were incubated for 10 min and shaken for 10 s before polarization was measured using a CLARIOstar Plus microplate reader with an excitation wavelength of 482/16 nm and an emission wavelength of 530/40 nm; dichroic mirror 504; flashes: 200. All data were analyzed using GraphPad Prism 7, and sigmoidal curves were fitted using the following Equation:

$$Y=\text{Bottom}+\left(\mathrm{Top}-\text{Bottom}\right)/\left(1+10^{\left(\left(\mathrm{Log}\;t_{1/2}-X\right)*\text{HillSlope}\right)}\right).$$

where *Y* = mP value, *X* = time, and Top and bottom = plateaus in mP.

The data were reported as the mean ± standard error of the mean.

### 4.4 Chymotrypsin‐coupled Pin1 PPIase assay

Pin1 (phosphorylated or non‐phosphorylated, 80 nM), in buffer containing 35 mM HEPES, pH 7.8, was incubated with 30 mg/mL ice‐cold chymotrypsin (diluted in 1 mM HCl) (Sigma‐Aldrich) and 2.5 mM of PPIase substrate (Suc‐AEPF‐pNA in 460 mM LiCl in trifluoroethanol) (#SAP‐3947‐PI, Vivitide) in a 1 mL cuvette. pNA release was measured at 4°C for 1 min on a Cary 300 UV–visible spectrophotometer using an absorption wavelength at 390 nm. To determine the relative enzymatic activity of the PPIase domain, the following formula was used:

$$\begin{array}{l} \text{Relative enzymatic activity}\;\left(\%\right)\\ \;=\left({\text{Abs}}_{\text{pPin}1}–{\text{Abs}}_{\text{blank}}\right)/\left({\text{Abs}}_{\text{Pin}1}–{\text{Abs}}_{\text{blank}}\right)*100. \end{array}$$

where Abs_pPin1_ corresponds to the reaction absorption detected at 0.5 min for phosphorylated Pin1 groups, Abs_Pin1_ represents the absorption detected at 0.5 min of non‐phosphorylated Pin1, and Abs_blank_, shows the reaction absorption detected at 0.5 min of a Pin1‐free reaction mixture.

The data were collected in triplicate and analyzed using GraphPad Prism 7. The data were reported as the mean ± standard error of the mean.

### 4.5 Native MS

To phosphorylate Pin1 for the native MS analysis, Pin1 (10 μM) was incubated at 30°C with 2 mM ATP, 10 mM MgCl_2_ (BDH Laboratory supplies), and 10,000 units PKA in 100 mM ammonium acetate pH 6.8 for 1, 3, 8, and 24 h. The concentration of PKA and Pin1 was optimized in order to detect sequential phosphorylation within the time course. At each time‐point, aliquots were taken, the reaction quenched by addition of EDTA (final concentration 20 mM), and the sample buffer exchanged using 3 kDa Amicon Ultra 0.5 mL centrifugal concentrators into 100 mM ammonium acetate pH 6.8 prior to MS analysis. Phosphorylation assays at each time‐point were carried out in triplicates, and the error was reported as the standard deviation.

Unlabeled Pintide [AcHN‐WFY(pS)PFLE‐CONH_2_] was purchased from China Peptides and dissolved in 100 mM ammonium acetate pH 8 to a final concentration of 1 mg/mL and stored at −80°C until use. To determine the affinity of Pin1 for differentially phosphorylated Pin1, Pintide was added to each Pin1 phosphorylation time‐point at a 2.5 μM: 25 μM Pin1 to Pintide ratio and the sample was directly infused into the mass spectrometer. Experiments were carried out in triplicates, and the error was reported as the standard deviation.

All native MS was performed on a QExactive HF mass spectrometer (Thermo Fisher Scientific) equipped with a nanoelectrospray ionization source that used gold‐coated borosilicate glass capillaries, pulled in‐house. Positive ionization mode was used throughout, with the capillary voltage set to 1.2 kV. The source temperature was set at 250°C, in source dissociation at 0, S‐lens RF at 100. The Pin1 concentration was set to 2.5 μM and a mass range of 1500–4000 m/z was acquired. Mass spectra were acquired using a maximum ion injection time of 100 ms. The automatic gain control was set to 1 x 10^6^ and the ions detected in the orbitrap with resolution set to 15,000. All data were analyzed using XCalibur (version 4.2). To quantify the abundance of unphosphorylated, phosphorylated Pin1 and Pin1‐Pintide complexes, the relative abundance of the peaks corresponding to the specific 9+, 8+, and 7+ Pin1 species of interest was summed and expressed as a percentage of all Pin1‐containing peaks detected.

### 4.6 Liquid‐chromatography MS

Phosphorylated Pin1 was digested with either trypsin (Promega) or the endoproteinase Lys‐C (Promega) at a 1:50 ratio of enzyme: protein. To localize the phosphorylation sites, trypsin digestion was performed. To quantify the phosphosite occupancy over the phosphorylation time‐course, differentially phosphorylated Pin1 was digested with Lys‐C. For both enzymatic reactions, Pin1 was digested in 100 mM ammonium bicarbonate pH 8.0 for 3 h at 37°C, and the reaction was quenched by the addition of formic acid (2% v/v final concentration). All peptides were separated using an UltiMate 3000 HPLC series, which was either coupled to a QExactive HF or Orbitrap Eclipse mass spectrometer (Thermo Fisher Scientific). Peptides were desalted on a PepMap100 C_18_ nanoViper trap column (75 μM x 2 cm, 3 μM particle size) (Thermo Fisher Scientific) and separated with a PepMap100 C_18_ nanoViper analytical column (75 μM x 15 cm, 3 μM particle size) (Thermo Fisher Scientific) using mobile phase A (H_2_O and 0.1% formic acid) and mobile phase B (100% acetonitrile and 0.1% formic acid) and eluted using a gradient from 3.2% A to 44% B over 30 min. The column flow rate was set to 350 nL/min with the column temperature set at 40°C. To quantify the extent of phosphorylation on Pin1, MS/MS was performed on the QExactive HF mass spectrometer. Full scan MS1 spectra were acquired in the Orbitrap mass analyzer using a resolution of 120,000 at m/z 200, automatic gain control of 3 x 10^6^, a maximum injection time of 50 ms, and a mass range between 380 and 1600 m/z. For MS/MS experiments, precursor ions were selected based on the Top 20 abundant ions. Precursor ions were isolated in the quadrupole with a 1.2 m/z window and subjected to higher‐energy collisional dissociation (HCD) with a normalized HCD energy of 28. The resolution was set to 15,000, with a maximum injection time of 50 ms and automatic gain control of 1 x 10^5^.

To prevent phospho‐loss resulting in misassignment of the phosphosite location (Boersema et al., 2009), electron transfer dissociation with supplemental HCD (EThcD) was performed on the trypsin‐digested peptides to localize the phosphosites. Precursor ions were selected based on the Top 15 abundant ions and isolated in the quadrupole with a 1.2 m/z window. ETD was performed using an automatically determined reagent time set based on precursor charge state. In all cases, a supplemental activation was applied using a normalized HCD energy of 10. Fragment ions were detected in the Orbitrap using a resolution setting of 30,000. The maximum injection time and automatic gain control were set to auto. For all MS/MS experiments, the dynamic exclusion was employed for 20 s on a single‐charge state per precursor, and only charge states from 2+ to 6+ were selected for MS/MS.

All RAW files were processed and analyzed using Proteome Discoverer version 2.5. (Thermo Fisher Scientific). The default settings were used unless otherwise stated. For all searches, enzyme cleavage was set to full, and the maximum number of missed cleavages set to 6 with a minimum peptide length of 6. A precursor mass tolerance of 10 ppm was used, with a fragment mass tolerance of 0.02 Da. When searching data for phosphorylated residues, the dynamic modifications included phosphorylation (+79.966 Da; S, T, Y) and oxidation (+15.995 Da) of methionine. The maximum number of modifications per peptide was set to 4. The peptide validator node was set to filter for a false discovery rate of 0.01. For PTM site localization, ptmRS was used (Taus et al., 2011). The PTM site probability threshold was set to 100, and site localization verified manually from the annotated ions in the raw MS2 spectra. Full sequence coverage of Pin1 was obtained. Additional phosphosites, Ser58, Ser65, and Ser67 (Table S4) were detected after 24 h of incubation with PKA, however, these were not consistently detected across all timepoints and, therefore, were excluded from phosphosite quantitation.

To quantify the abundance of phosphorylated peptides, the retention time of the eluted peptides was identified using Proteomic Discoverer v.2.5. Spectra were averaged across the peptides chromatographic peak, and the percentage of phosphorylated peptide was calculated as a percentage of the total abundance of unphosphorylated and phosphorylated peptide. Full details of the peptides used for quantitation are detailed in the Supporting Information (Table S4).

## AUTHOR CONTRIBUTIONS

**Danielle F. Kay:** Validation; visualization; writing – review and editing; writing – original draft; data curation. **Adem Ozleyen:** Writing – review and editing; visualization; validation; data curation. **Cristina Matas De Las Heras:** Data curation; visualization; validation; writing – review and editing. **Richard G. Doveston:** Conceptualization; writing – review and editing; supervision; funding acquisition. **Aneika C. Leney:** Conceptualization; data curation; visualization; validation; writing – original draft; writing – review and editing; funding acquisition; supervision.

## CONFLICT OF INTEREST STATEMENT

The authors declare that they have no conflicts of interest with the contents of this article.

## Supporting information


**DATA S1.** Supporting Information.

## Data Availability

All data within this manuscript are freely available from the University of Birmingham data archive at https://doi.org/10.25500/eData.bham.00001101.
