# Supplementary material for: Dissecting the functional behavior of the differentially phosphorylated prolyl isomerase, Pin1
Source: Protein Sci. 2024 Aug 16;33(9):e5138. doi: 10.1002/pro.5138 (PMC11328113; doi:10.1002/pro.5138)
Supplement: Supplementary file 1 — DATA S1. Supporting Information. [file PRO-33-e5138-s001.docx]

**Supplementary Material**

**Dissecting the functional behaviour of the differentially phosphorylated prolyl isomerase, Pin1**

Danielle F. Kay^a^, Adem Ozleyen^b,c^, Cristina Matas De Las Heras^b,c^, Richard G. Doveston^b,c^, Aneika C. Leney^a^ *

**Contents**

Figure S1. Fluorescence polarisation controls……………………………………………………………….1

Figure S2. Localisation of Ser16 phosphosite………………………………………………………………..2

Figure S3. Localisation of Ser71 phosphosite………………………………………………………………...2

Figure S4. MS of phosphorylated peptides upon incubation of Pin1 with PKA……………………………3

Figure S5. Impact of phosphorylation on Pin1 substrate recognition…………………………………….…4

Figure S6. Pin1 protein sequence………………………………………………………………………….…..5

Table S1. Theoretical and observed masses for Pin1 proteoforms and their complexes……………….5

Table S2. Peptides used to quantify phosphorylation……………………………………………………….5

Table S3. Quantification of Ser16 and Ser71 phosphorylation sites……………………………………….5

Table S4. Phosphorylation stoichiometry for all detected phosphorylated peptides………………………6

Table S5. Quantification of Pintide bound to each Pin1 proteoform………………………………………..6

**
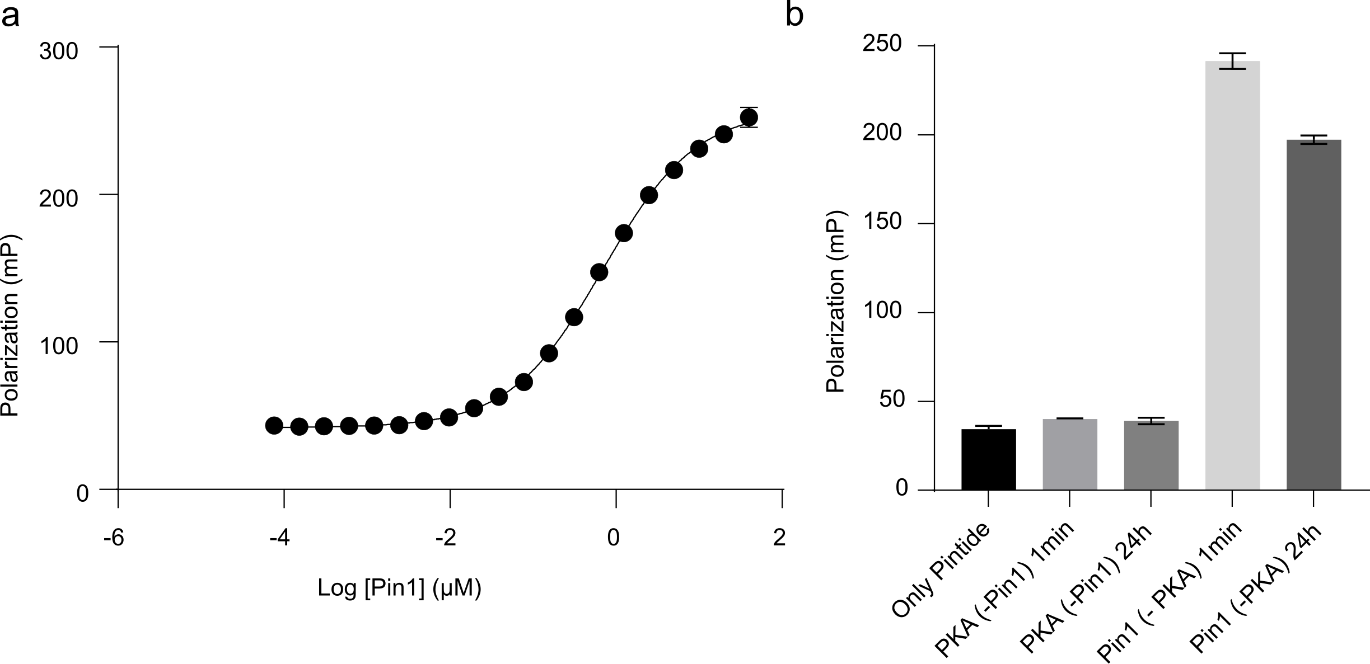
**

**Figure S1.** (a) Fluorescence polarisation data for Pin1 titration to fluorescently labelled Pintide (10 nM). The apparent Kd was 0.7 µM. The data was fitted to a variable slope sigmoidal least squares model in GraphPad Prism. (b) Fluorescence polarisation control data showing polarisation, mP values, for the fluorescently labelled Pintide alone (Only Pintide, black), PKA in the presence of Pintide without Pin1 at 1 min and 24 h time points, and Pin1 in the presence of Pintide without PKA at 1 min and 24 h time points. Data was collected in triplicates and presented as the mean ± standard error of the mean.


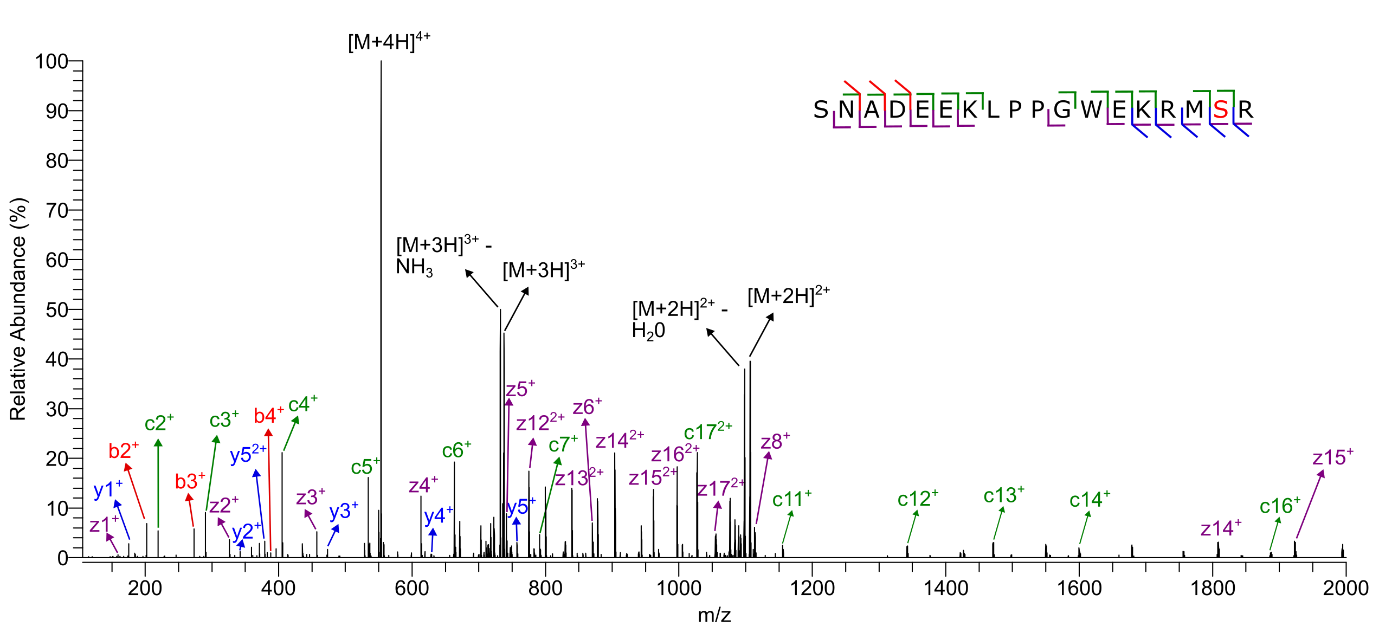
**Figure S2. Bottom-up mass spectrometry localised the site of phosphorylation on the WW domain to Ser16.** Digestion carried out with trypsin. a) MS/MS spectrum of precursor phosphorylated peptide SNADEEKLPPGWEKRMSR (residues 1-18) fragmented with EThcD. The phosphorylated residue is highlighted in a red in the sequence. Spectra were taken from a 24 hour time-point. y ions are annotated in blue, b ions are annotated in red, c ions are annotated in green and z ions are annotated in purple. Precursor peak is [M+4H]^4+^. Charge reduced peaks are also labelled.


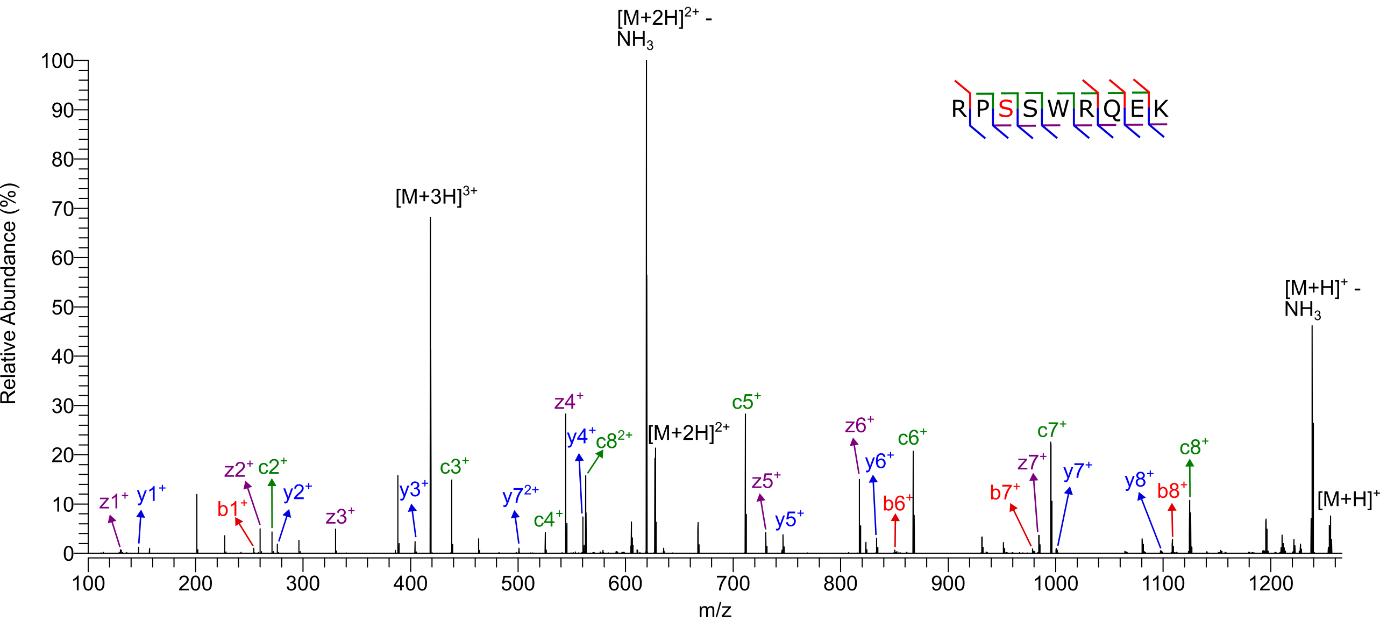


**Figure S3. Bottom-up mass spectrometry localised the site of phosphorylation on the PPIase domain to Ser71.** Digestion carried out with trypsin. a) MS/MS spectrum of precursor phosphorylated peptide RPSSWRQEK (residues 69-77) fragmented with EThcD. The phosphorylated residue is highlighted in a red in the sequence. Spectra were taken from a 24 hour time-point. y ions are annotated in blue, b ions are annotated in red, c ions are annotated in green and z ions are annotated in purple. Precursor peak is [M+3H]^3+^. Charge reduced peaks are also labelled.

**
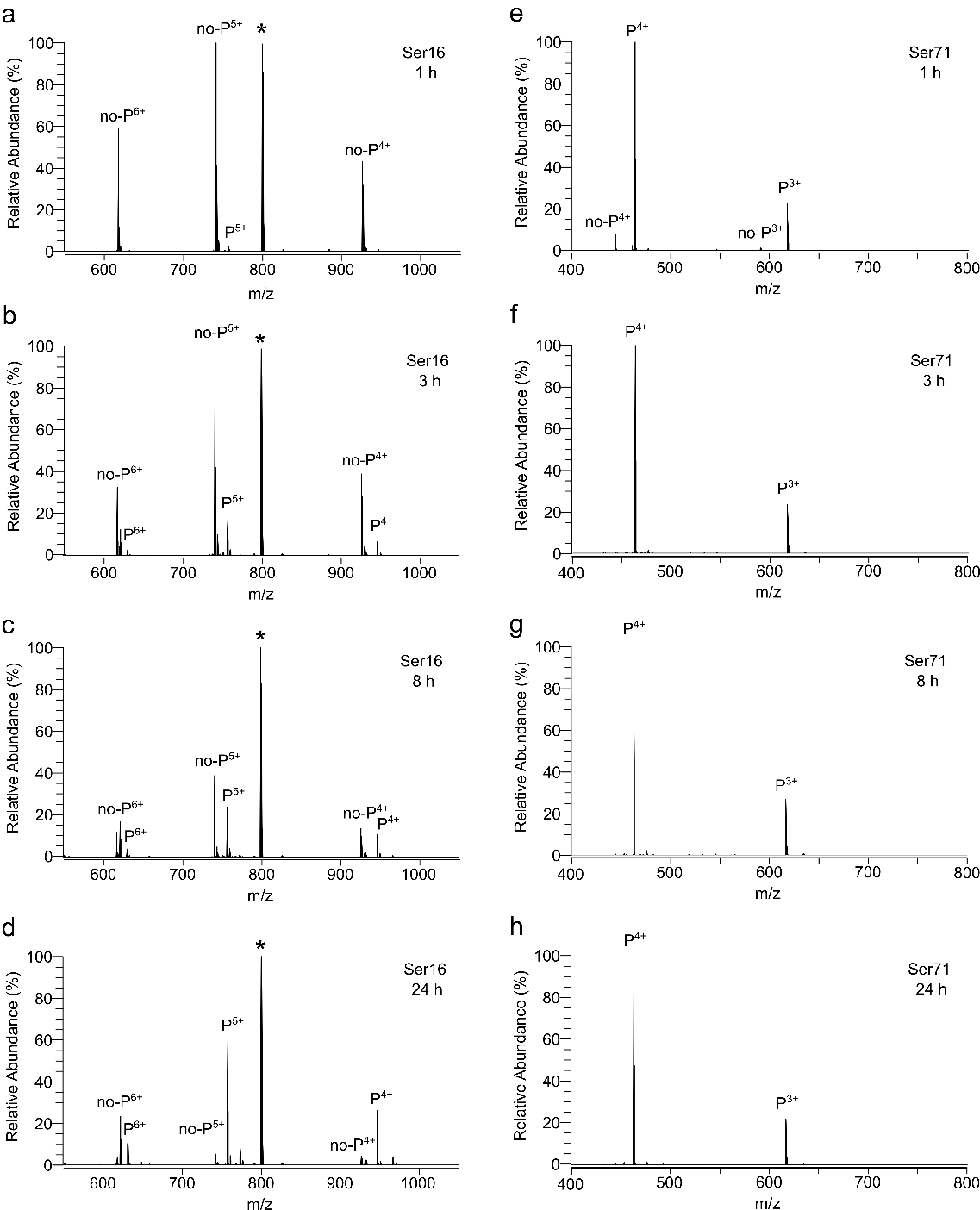
**

**Figure S4. Example mass spectra showing the abundance of phosphorylated peptides upon incubation of Pin1 with PKA.** The MS data was obtained from extracted ion chromatograms covering 15.0-17.0 min, and 12.1-13.4 min for Ser 16 (a-d), and Ser71 (e-h), respectively, after 1 h (a,e), 3 h (b, f), 8 h (c, g) and 24 h (d, h) incubation of Pin1 with PKA. The * corresponds to a co-eluting Pin1-associated peptide.


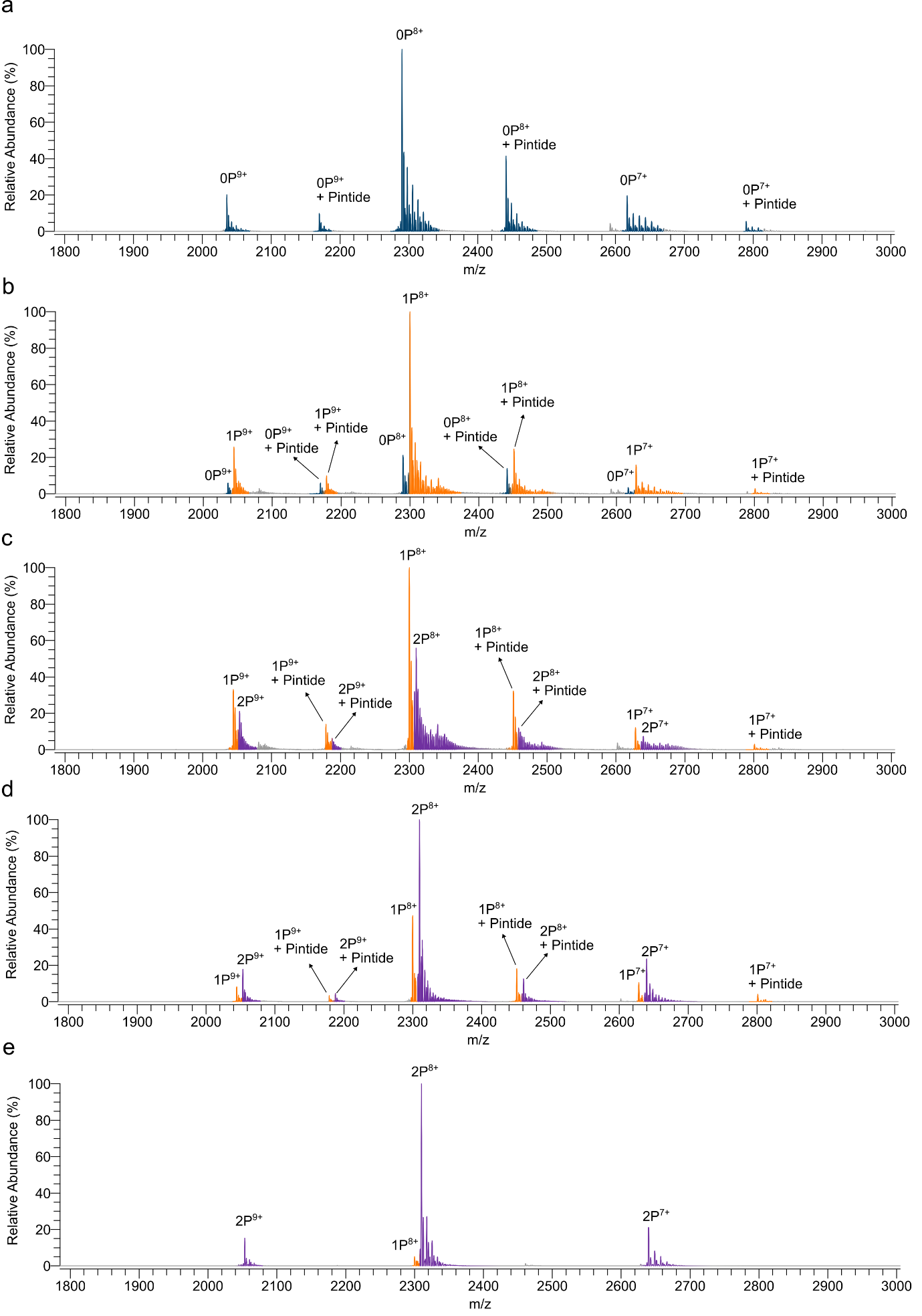


**Figure S5.** **Native MS show impact of phosphorylation on Pin1 substrate recognition.** Native MS of Pintide incubated with (a) unmodified Pin1, and Pin1 after phosphorylation with PKA for (b) 1 h, and (c) 3 h, d) 8 h, e) 24 h. Peaks corresponding to the 9+, 8+ and 7+ charge state of unmodified Pin1, singly phosphorylated Pin1 and doubly phosphorylated Pin1 are highlighted in blue, orange and purple, respectively.

*
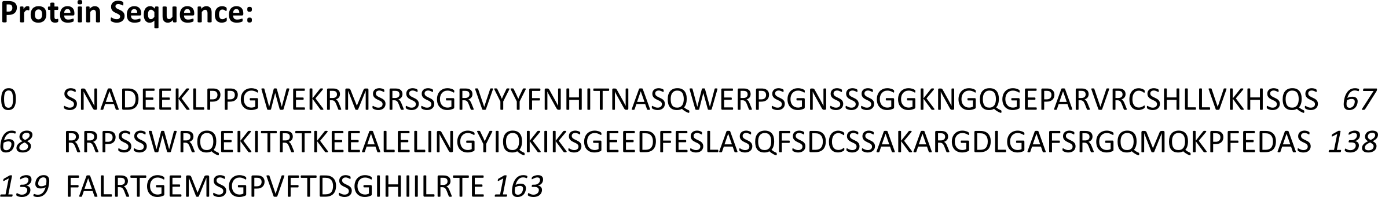
*

**Figure S6. Pin1 protein sequence.** Note the residue numbers have been shifted -1 to match the literature. i.e. Ser17 in the above sequence is labelled as Ser16 in the main text.

**Table S1. Theoretical and observed masses for Pin1 proteoforms and their complexes.**

| **Protein** | **Theoretical Mass (Da)** | **Measured Mass (Da)** |
| --- | --- | --- |
| Pin1 unphosphorylated | 18313.3 | 18311.7 ± 0.1 |
| Pin1 singly phosphorylated | 18393.3 | 18391.4 ± 0.5 |
| Pin1 doubly phosphorylated | 18473.3 | 18471.7 ± 0.1 |
| Pin1 unphosphorylated + Pintide | 19522.8 | 19520.8 ± 0.3 |
| Pin1 singly phosphorylated + Pintide | 19602.8 | 19601.1 ± 0.2 |
| Pin1 doubly phosphorylated + Pintide | 19682.8 | 19680.7 ± 0.4 |

**Table S2. Peptides used to quantify Ser 16 and Ser71 phosphorylation.**

| **Peptide sequence** | **Phosphorylated residue** | **Observed m/z value** | **Charge states** |
| --- | --- | --- | --- |
| RMSRSSGRVYYFNHITNASQWERPSGNSSSGGK | N/A | 618.13, 741.56, 926.70 | 6+, 5+, 4+ |
| RMpSRSSGRVYYFNHITNASQWERPSGNSSSGGK | Ser16 | 631.46, 757.55, 946.69 | 6+, 5+, 4+ |
| HSQSRRPSSWRQEK | N/A | 442.98. 590.30, | 4+, 3+ |
| HSQSRRPpSSWRQEK | Ser71 | 462.97, 616.96 | 4+, 3+ |

**Table S3. Quantification of Ser16 and Ser71 phosphorylation as determined by LC-MS/MS.**

| **Time (hours)** | **Phosphorylation Site** | | **Combined Phosphorylation Stoichiometry** |
| --- | --- | --- | --- |
|  | **Ser16** | **Ser71** |  |
| 1 | 0.04 | 0.91 | 0.95 |
| 3 | 0.14 | 1.00 | 1.14 |
| 8 | 0.40 | 1.00 | 1.4 |
| 24 | 0.84 | 1.00 | 1.84 |

**Table S4. Phosphorylation stoichiometry for additional phosphorylated Pin1 peptides detected by LC-MS/MS following trypsin digestion.** Fragmentation was performed using EThcD. The peptides detected containing phosphorylation sites are shown along with their unmodified counterparts used for quantification. In the case where two phosphorylation sites were detected, the quantified unphosphorylated component includes the adjacent singly phosphorylated sites.

| **Phosphorylated residue(s)** | **Peptide sequences used for quantification** | | **Phosphorylation Stoichiometry (24h)** |
| --- | --- | --- | --- |
|  | **Unphosphorylated** | **Phosphorylated** |  |
| Ser58 | C_57_SHLLVK_63_  V_55_RCSHLLVK_63_  NGQGEPARVRCSHLLVK C_57_SHLLVKHpSQSR | V_55_RCpSHLLVK_63_  V_55_RCpSHLLVKHpSQSR_68_ | 0.19 |
| Ser65 | H_64_SQSRRPpSSWR_74_  H_64_SQpSRRPpSSWR_74_  H_64_SQSRRPpSSWRQEK_77_  H_64_SQpSRRPpSSWRQEK_77_ | V_55_RCpSHLLVKHpSQSR_68_  C_57_SHLLVKHpSQSR_68_ | 0.05 |
| Ser67 | H_64_SQSRRPpSSWR_74_  H_64_SQSRRPpSSWRQEK_77_ | H_64_SQpSRRPpSSWR_74_  H_64_SQpSRRPpSSWRQEK_77_ | 0.05 |

**Table S5. Quantification of Pintide bound to each Pin1 proteoform.** SD represents the standard deviation between triplicate measurements, whereby * denotes p <0.05, ** denotes p <0.01 as determined by one-way ANOVA with post-hoc Tukey HSD test.

| **Number of phosphosites** | **% Pintide bound (±SD)** |
| --- | --- |
| 0 | 39 ± 5 |
| 1 | 26 ± 6* |
| 2 | 4 ± 1** |
